# High-Performance ZIF-7@PANI Electrochemical Sensor for Simultaneous Trace Cadmium and Lead Detection in Water Samples: A Box–Behnken Design Optimization Approach

**DOI:** 10.3390/s25051336

**Published:** 2025-02-22

**Authors:** Sondes Guesmi, Nashwan H. Ali, Nadhem Missaoui, Zouhaier Aloui, Chama Mabrouk, Carlos Castilla Martinez, Fraj Echouchene, Houcine Barhoumi, Nicole Jaffrezic-Renault, Hamza Kahri

**Affiliations:** 1 Laboratory of Interfaces and Advanced Materials, University of Monastir, Monastir 5000, Tunisiamissaoui.nadhem1@gmail.com (N.M.); houcine.barhoumi@fsm.rnu.tn (H.B.); 2 Apllied Chemistry Department, Applied Sciences Collage, University of Samarra, Samarra 34010, Iraq; 3 Chemistry Department, College of Science, King Khalid University (KKU), P.O. Box 9004, Abha 61413, Saudi Arabia; 4 Institut Européen des Membranes, IEM—UMR 5635, University of Montpellier, CNRS, 34090 Montpellier, France; 5 University of Sousse, Higher Institute of Applied Sciences and Technology of Sousse, Cité Ettafala, Ibn Khaldoun, Sousse 4003, Tunisia; 6 Laboratory of Electronics and Microelectronics LR99ES30, Faculty of Sciences, University of Monastir, Monastir 5000, Tunisia; 7 Institute of Analytical Sciences, University of Lyon, 69622 Villeurbanne, France

**Keywords:** ZIF-7@PANI composite, electrochemical sensor, heavy metal detection, Box–Behnken design, cadmium and lead sensing

## Abstract

**Highlights:**

**What are the main findings?**

- Development of an innovative sensor based on ZIF-7 and polyaniline for the simultaneous detection of cadmium (Cd²⁺) and lead (Pb²⁺) ions in water.
- Optimization of the sensor’s performance using the Box-Behnken design (BBD), leading to enhanced sensitivity and selectivity.

**What is the implication of the main finding?**

- The ZIF-7@PANI/GCE sensor demonstrated low detection limits (2.96 nM for Pb²⁺ and 10.6 nM for Cd²⁺), making it highly suitable for trace-level heavy metal detection.
- The successful validation of the sensor using real water samples highlights its potential for practical applications in environmental monitoring and water quality assessment.

**Abstract:**

This study presents the development of an innovative electrochemical sensor for the simultaneous detection of cadmium (Cd^2+^) and lead (Pb^2+^) ions in environmental samples. The sensor is developed based on a composite material of zeolite imidazolate framework ZIF-7 and polyaniline (PANI), referred to as ZIF-7@PANI, where ZIF-7 is rapidly synthesized at room temperature and polyaniline used to enhance the conductivity of the composite. Characterization via X-ray diffraction (DRX), scanning electron microscopy (SEM), and Fourier transform infrared spectroscopy (FTIR) confirmed successful synthesis. The composite was applied to a glassy carbon electrode (GCE) using drop-casting for heavy metal ion detection. Experimental parameters—including pH, incubation time, deposited quantity, and drying time—were optimized using the Box–Behnken design. Under optimal conditions, the ZIF-7@PANI/GCE sensor demonstrated a broad dynamic concentration range (0.002–1 µM for Pb^2+^ and 0.02–30 µM for Cd^2+^), with low detection limits (2.96 nM for Pb^2+^ and 10.6 nM for Cd^2+^). It also exhibited strong anti-interference properties and high recovery rates (85–110%), highlighting its potential for real practical applications.

## 1. Introduction

Water scarcity, exacerbated by climate change—particularly global warming—poses a significant challenge for emerging countries [1]. Among the many concerns, heavy metal contamination in water reservoirs stands out [2], primarily caused by industrial activity and agriculture. This issue impacts billions of people worldwide who lack access to clean and safe drinking water [3]. Lead (Pb^2+^) and cadmium (Cd^2+^) ions, in particular, pose severe health risks, contributing to lead poisoning, digestive issues, increased cancer susceptibility (notably breast cancer), and, in acute cases, abdominal muscle cramps and toxic hepatitis [4,5]. Traditional analytical methods for detecting heavy metal ions in water, such as atomic emission spectroscopy (AES) [6], atomic absorption spectroscopy (AAS) [7], X-ray fluorescence spectrometry [8], and inductively coupled plasma mass spectrometry (ICP-MS) [9], are often expensive and time-consuming. Therefore, developing sensitive sensors capable of simultaneous, real-time detection of Cd^2+^ and Pb^2+^ ions is essential. Recent advancements in technology have significantly improved detection capabilities, particularly in enhancing both sensitivity and selectivity for environmental monitoring applications [10]. Electrochemical detection offers an ideal solution due to its cost-effectiveness, user-friendly operation, high sensitivity, and compatibility with portable testing equipment [11,12]. Electrode materials play a key role in electrochemical sensor performance [13], leading to extensive research aimed at improving their efficiency. Metal-organic frameworks (MOFs) have attracted considerable attention due to their large specific surface area, abundant adsorption sites, and tunable functionality [14]. They have been widely applied in catalysis [15], gas storage [16], separation and purification processes [17], drug delivery [18], and environmental remediation [19]. More recently, MOFs have shown great promise in developing highly sensitive electrochemical sensors for heavy metal detection, contributing to water quality monitoring [20,21]. Nonetheless, MOF-based electrodes often suffer from limitations such as low conductivity, challenges in large-scale preparation, and poor repeatability. To address these issues, researchers have explored incorporating conductive materials into MOFs, including metals [22], carbon nanomaterials [23,24], biopolymers [25], and polymers [26].

Zeolitic Imidazolate Frameworks (ZIFs), a subclass of MOFs, are known for their tetrahedral structure, formed through strong bonds between metal ions and four imidazolate ligands [27]. Various methods have been reported for producing ZIFs, including solvothermal [28], sol-gel [29], surfactant-assisted methods [30], and microwave/ultrasound-assisted techniques [31]. However, these methods often demand long reaction times due to slow interactions, highlighting the need for high-performance, eco-friendly, and easily accessible materials with controlled crystal properties. Templates, like polymers or surfactants, offer a way to achieve ZIFs with controlled morphology, preventing non-uniform agglomeration. Growing interest in room-temperature synthesis has led to more efficient approaches, such as the polyethylene glycol (PEG)-templated method introduced by Nadhem et al. for producing highly microporous nanoZIF-8 [32,33].

In this study, a PEG-based soft template was used to rapidly and easily synthesize porous nano-ZIF-7. This material was then combined with polyaniline (PANI) to form a ZIF-7@PANI composite, which was applied as a sensitive layer for the simultaneous electrochemical detection of Cd^2+^ and Pb^2+^ ions. Key experimental parameters were optimized using the Box–Behnken design to improve the sensor’s electroanalytical performance. This study focused on the investigation of sensitivity, anti-interference properties, and their applicability to real samples for the detection of Cd^2+^ and Pb^2+^ ions in aqueous solutions.

## 2. Experimental

### 2.1. Chemicals

Zn(NO_3_)_2_·6H_2_O, benzimidazole (blm; C_7_H_6_N_2_; 98%), polyethylene glycol (PEG, 20,000 g/mol), dimethylformamide (DMF, 99%), methanol (99%), triethylamine (TEA; (C_2_H_5_)_3_N; 99%), and aniline (99%) were purchased from Sigma-Aldrich (Darmstadt, Germany). (NH_4_)_2_S_2_O_8_ (APS), Pb(NO_3_)_2_·6H_2_O, and Cd(NO_3_)_2_·6H_2_O were obtained from Fluka Chemika (Buchs, Switzerland). The acetate buffer was used to dissolve Pb(NO_3_)_2_ and Cd(NO_3_)_2_·3H_2_O, resulting in the preparation of the stock solution of Pb^2+^ and Cd^2+^ ions.

### 2.2. Preparation of ZIF-7@PANI Nanocomposite

#### 2.2.1. Synthesis of Nano-ZIF-7

Nano-ZIF-7 was synthesized following a methodology similar to a previously reported publication for nanocrystalline ZIF-8 [32]. Zn(NO_3_)_2_·6H_2_O and blm served as precursors, while PEG (20,000 g/mol) acted as a soft template. In the initial step, 400 mg of PEG powder was dispersed in 20 mL of DMF, followed by the addition of Zn(NO_3_)_2_·6H_2_O (2.5 g, 8.40 mmol) with mild stirring for 2 min to form solution A. Separately, benzimidazole (3.08 g, 26.07 mmol) was dispersed in DMF (20 mL), and TEA, employed as a base, was gradually added (7.26 mL) under stirring over 2 min to prepare solution B. Upon complete dissolution, solution A was slowly added to solution B and stirred for 5 min at room temperature. The resulting white precipitated was separated by filtration, rinsed with ethanol to remove residual reagents (including PEG), and collected via centrifugation (4000 rpm, 15 min). Finally, the obtained white material was dried in a vacuum oven at 150 °C for 24 h, yielding the final ZIF-7 product.

#### 2.2.2. Synthesis of ZIF-7@PANI Nanocomposite

The ZIF-7@PANI nanocomposite was synthesized according to a prior report [26]. Initially, 0.3 g of ZIF-7 was dispersed in 15 mL of 1 M HCl via sonication. Subsequently, 30 μL of aniline was added to the mixture, which was then sonicated for 20 min. Following this, 0.114 g of APS in 5 mL of 1 mol L^−1^ HCl was added dropwise to the mixture at 0 °C, and then the mixture was stirred overnight to ensure thorough polymerization. The resulting solid was washed three times alternately with distilled water and ethanol via centrifugation then dried at 60 °C for 24 h. A similar method was used to synthesize pristine PANI by polymerizing aniline (30 μL) in 15 mL of 1 mol L^−1^ HCl, followed by the addition of APS, without incorporating ZIF-7.

### 2.3. Preparation of Modified Electrodes

Before measurements, the surface of the glassy carbon electrode (GCE) was carefully polished with 0.3 µm alumina powder then immersed in an ethanol–water solution and sonicated for 2 min to ensure thorough cleaning. To modify the electrode surface, a homogeneous suspension of the nanocomposite was prepared by dispersing 2 mg of ZIF-7@PANI in 1 mL of distilled water. From this suspension, a 10 μL was drop-costed onto the electrode surface and left to dry for 3 h. The ZIF-7/GCE and PANI/GCE electrodes were prepared using the same procedure by drop-casting the ZIF-7 and PANI suspensions, respectively, onto the electrode surface for comparative electrochemical analysis.

### 2.4. Characterization Methods

The morphological and structural properties of PANI, ZIF-7, and ZIF-7@PANI were investigated using various techniques. X-ray diffraction (XRD) analysis was carried out using a Bruker D8 Discover diffractometer (Billerica, MA, USA), while Fourier transform infrared spectroscopy (FT-IR) spectra were obtained employing a Perkin Elmer Spectrum 100 spectrometer (Waltham, MA, USA) featuring a universal ATR sampling accessory. Scanning electron microscopy (SEM) imaging was performed with a Supra 40VP Field Emission microscope (Oberkochen, Germany).

### 2.5. Electrochemical Tests

Electrochemical measurements were conducted using a three-electrode configuration within an electrochemical cell. A glassy carbon electrode (diameter: 3 mm) was used as the working electrode, while an Ag/AgCl/KCl electrode and a platinum wire functioned as the reference and counter electrodes, respectively. Electrochemical techniques, including electrochemical impedance spectroscopy (EIS), cyclic voltammetry (CV), and differential pulse voltammetry (DPV), were performed using an Autolab PGSTAT 320N Potentiostat (Metrohm, Milan, Italy), controlled via NOVA 1.5 software for data analysis. Metal ion accumulation was carried out under open circuit conditions by immersing the modified electrode in a stirred buffer solution containing varying metal ion concentrations for an optimized accumulation period. To investigate the electrochemical behavior of the prepared electrodes, cyclic voltammetry (CV) and electrochemical impedance spectroscopy (EIS) were conducted. These analyses were performed in a 5 mM ferrocyanide (Fe(CN)_6_^−3/−4^) solution as probe in 0.1 M KCl.

### 2.6. Water Sample Analysis

Seawater samples were obtained from the SKANES region in the city of Monastir, Tunisia. The seawater supernatant, along with tap and mineral water, was diluted twenty-fold for electrochemical detection without prior treatment. The concentrations of heavy metal ions were assessed by using a standard calibration curve, and the accuracy was verified by adding standard solution to the actual water sample solution.

## 3. Results and Discussions

### 3.1. Structural and Morphological Characterization

**Figure 1:**
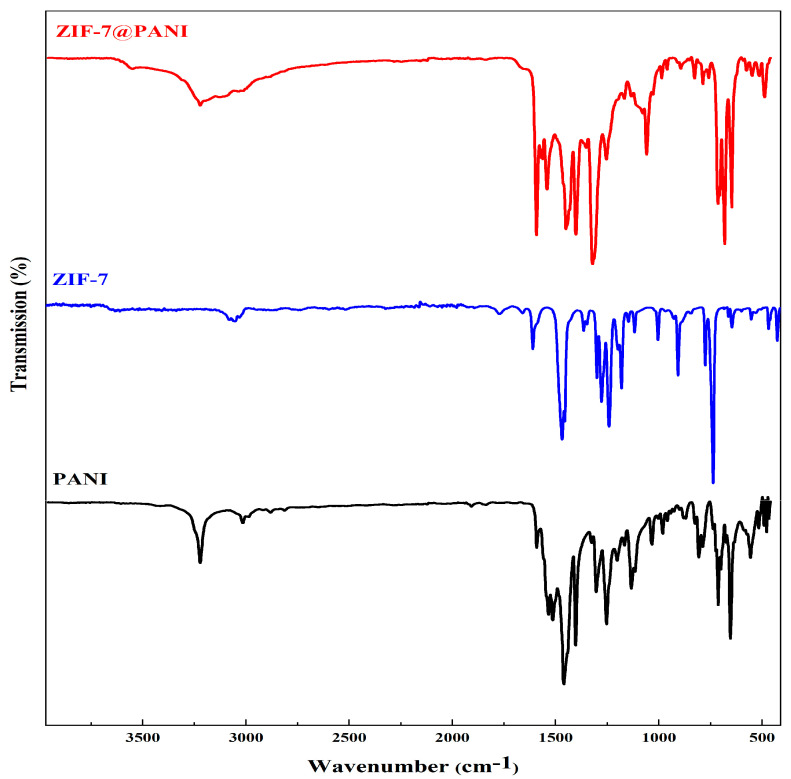
FTIR spectra of PANI, synthesized ZIF-7 and ZIF-7@PANI composite.

The chemical composition of ZIF-7 was confirmed by FTIR analysis (Figure 1). Spectral features between 600 and 1500 cm^−1^ correspond to the bending and stretching modes of the benzimidazole ring structure [34]. Specifically, the peaks observed at 750 and 1455 cm^−1^ were attributed to C-H and C=C bonds, respectively [35]. The stretching of =C-H in aromatics appeared at 3032 and 3068 cm^−1^, while the C-C stretching of the aromatic ring was observed at 1465 and 1611 cm^−1^ [36]. Additionally, the band at 422 cm^−1^ was associated with the Zn-N stretching vibration [37]. These findings confirm the successful coordination between benzimidazole and Zn^2+^ in the ZIF-7 nanoparticles.

The FTIR spectrum of polyaniline (PANI) shows absorption peaks at 1560, 1472, 1297, and 1117 cm^−1^. The peaks observed at 1560 and 1472 cm^−1^ are associated with the C=C stretching deformations of the quinoid and benzene rings, indicating the presence of emeraldine salt state in PANI. The band at 1297 cm^−1^ is assigned to the C–N stretching vibration, while the band at 1117 cm^−1^ is attributed to the quinonoid unit in doped PANI [38]. In the ZIF-7@PANI spectrum, the peak at 1117 cm^−1^ experiences a blue shift to 1135 cm^−1^, attributed to the strong electrostatic interaction between PANI and ZIF-7. Additionally, the C=C elongation of the quinoid ring and benzene rings in ZIF-7@PANI shifts from 1560 and 1472 cm^−1^ in pure PANI to 1570 and 1493 cm^−1^, suggesting enhanced π-π interactions between the ZIF-7 framework and PANI [39].

**Figure 2:**
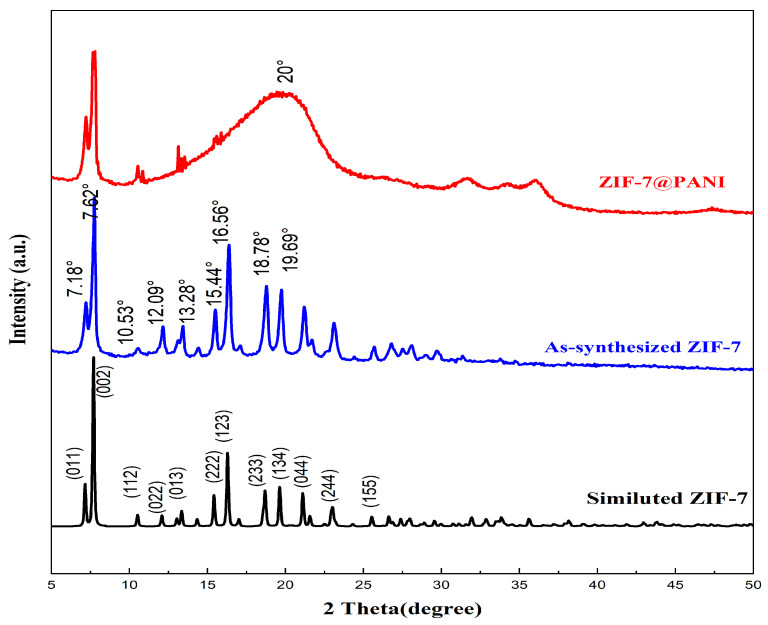
The powder X-ray diffraction patterns of the ZIF-7 and ZIF-7@PANI.

The three samples were analyzed by the powder XRD technique. The strong similarity between the simulated and synthesized XRD patterns suggests the production of pure ZIF-7, free from impurities or additional phases (Figure 2). Indexing of the diffractogram reveals significant alignment with the corresponding lattice parameters and space group (R-3). Notably, the observed broadening of the diffraction peaks indicates the formation of smaller ZIF-7 crystals. Figure 2 displays the XRD patterns for the ZIF-7@PANI composite, with peaks at 7.18°, 7.62°, 10.53°, 13.28°, 15.44°, and 19.69° (in 2θ), confirming the presence of ZIF-7 crystals with a cubic structure (R-3) [34,36,40]. Additionally, the peak at 20° confirms the inclusion of PANI in the composite, validating the presence of semi-crystalline PANI planes [41].

**Figure 3:**
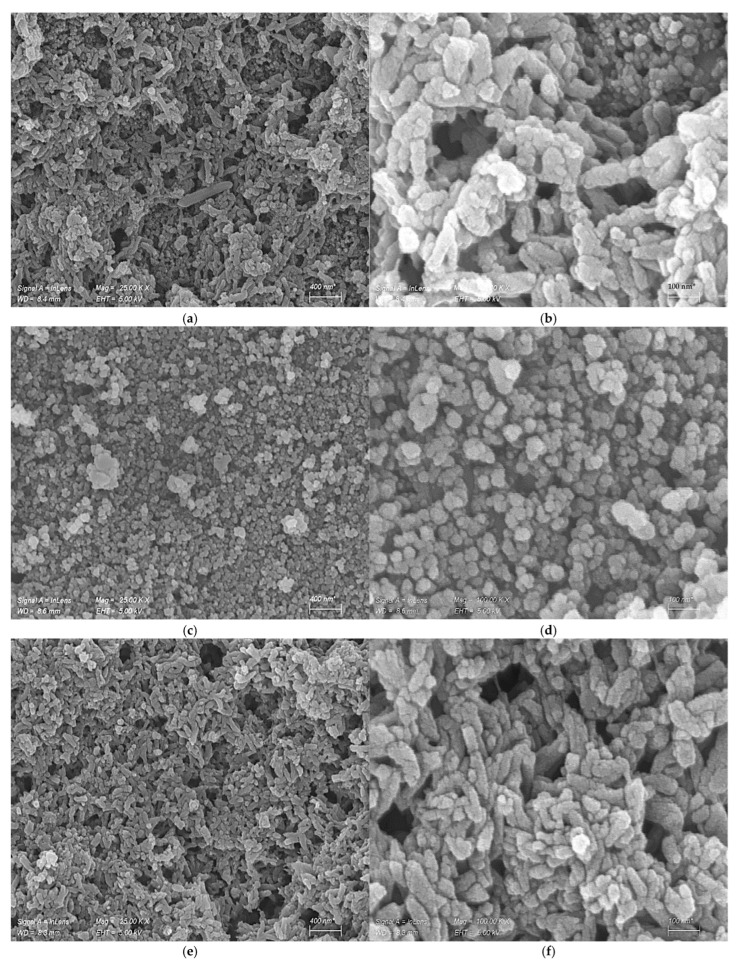
SEM images of PANI/SPCE (**a**,**b**), ZIF-7/SPCE (**c**,**d**), and ZIF-7@PANI/SPCE (**e**,**f**). Images (**a**,**c**,**e**) are taken at 25,000× magnification with a scale bar of 400 nm, while images (**b**,**d**,**f**) are taken at 100,000× magnification with a scale bar of 100 nm.

Figure 3 shows the SEM images of the different modified screen-printed electrodes (SPCE) constructed using ZIF-7, PANI, and the composite ZIF-7@PANI.

The SEM image of the PANI-modified electrode reveals a non-uniform topography and a spongy morphology (Figure 3a,b). The SEM image of the pure ZIF-7 matrix deposited on the SPCE surface shown in Figure 3c,d displays well-defined octahedral and cubic nanoparticles, ranging in size from 20 nm to 60 nm. After the incorporation of pure ZIF-7 into the polymer, noticeable deformations in the morphology of the composite surface are observed (Figure 3e,f). This change can be attributed to the interlinking between PANI and the pure ZIF-7 matrix. The SEM images in Figure 3e,f strongly indicate the successful formation of the ZIF-7@PANI composites, with small octahedral and cubic crystals intertwined. The resulting composite sample also displays a significant degree of agglomeration.

**Scheme 1:**
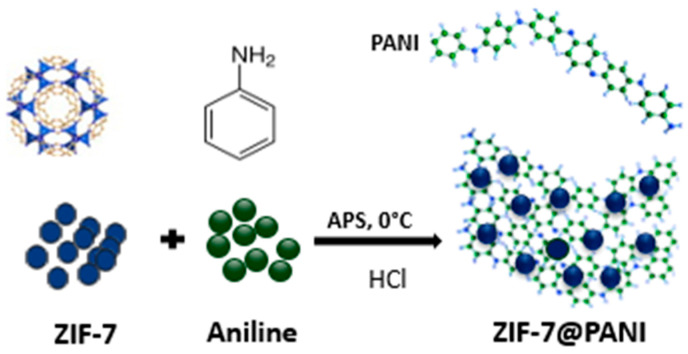
Schematic illustration of ZIF-7@PANI composite synthesis.

Following the interpretation of the results from the characterization techniques, the synthesis process of the ZIF-7@PANI composite is illustrated in Scheme 1.

### 3.2. Electrochemical Characterization of the Modified Electrodes

**Figure 4:**
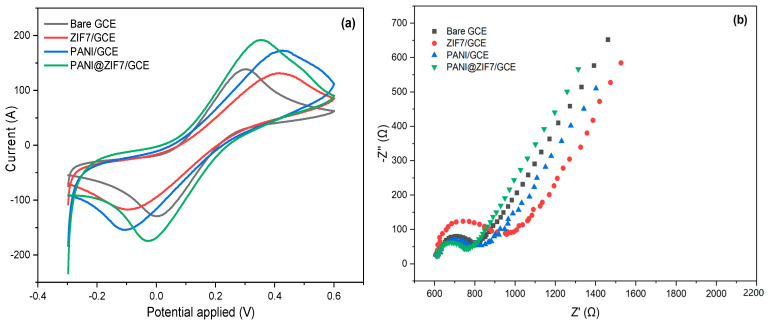
(**a**) CV and (**b**) EIS curves of bare GCE, ZIF-7/GCE, PANI/GCE, and ZIF-7@PANI/GCE in 0.1 M KCl solution containing 5 mM Fe(CN)_6_^−3/−4^.

As shown in Figure 4a, the cyclic voltammogram (CV) of the bare GCE exhibited clear and reversible redox peaks. After functionalizing the working electrode surface with the ZIF-7 matrix, a slight reduction in the electron transport rate was observed at the modified electrode interface, leading to a decrease in peak current compared to the bare electrode. This reduction can be attributed to the relatively low conductivity of ZIF-7, as discussed by Butler et al. (2017) [42]. In contrast, the PANI-modified electrode displayed increased current, due to the conductive nature and fast charge transfer capabilities of polyaniline. Notably, the PANI@ZIF-7-modified electrode displayed the highest current for redox peaks, indicating that combining ZIF-7 and PANI into a mixed matrix enhances electron transfer at the electrode surface. This improvement is due to the high conductivity of PANI and the considerable specific surface area of ZIF-7, making it a suitable choice as an electrochemical sensing platform.

In addition to CV, EIS was employed to obtain additional electrochemical information on the modified electrodes. Under a 0.2 V applied potential, EIS parameters were measured across a frequency range from 1 × 10^−3^ to 1 × 10 ^5^ Hz with a 5 mV amplitude. The EIS spectra of different modified GCEs are shown in Figure 4b, featuring both semicircular and linear sections, with the fitting circuit model provided in the inset. The unmodified GCE exhibited a relatively small R_ct_ value of approximately 190 Ω. For the ZIF-7/GCE, the R_ct_ increased to 295 Ω due to its mesoporous structure, which hinders the charge transfer process due to the low conductivity of the ZIF-7 matrix. Conversely, the R_ct_ at the PANI/GCE interface was lower than that of the bare electrode, attributed to the enhanced electrical conductivity of polyaniline. On the ZIF-7@PANI/GCE, the R_ct_ decreased to 140 Ω, suggesting that the ZIF-7 and PANI mixed matrix efficiently coated the surface, creating a porous and highly conductive structure with a significant active surface area.

**Figure 5:**
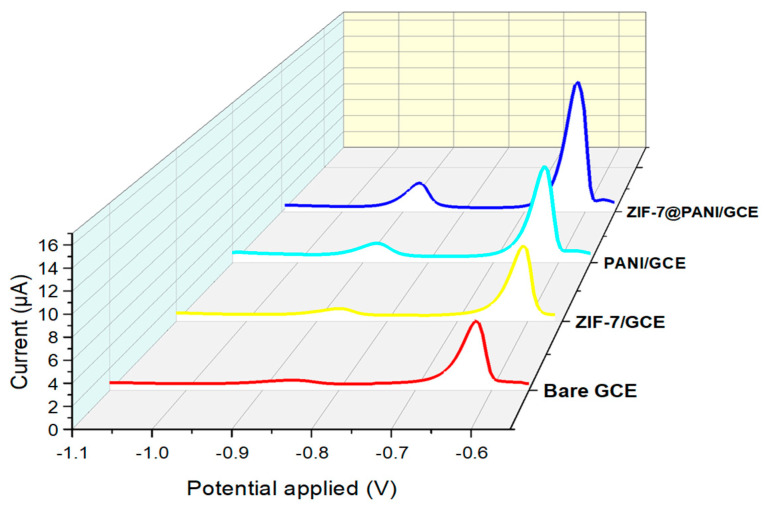
DPV curves of a 10 µM mixture of Cd^2+^ and Pb^2+^ ions in 0.1 m acetate buffer (pH 5) using different sensors.

The feasibility of the ZIF-7@PANI/GCE sensing electrode was evaluated for the simultaneous electrochemical determination of Cd^2+^ and Pb^2+^ ions in the same solution. DPV was employed to investigate the electrochemical response of 10 µM of both Cd^2+^ and Pb^2+^ using various modified electrodes. As depicted in Figure 5, the electrochemical behavior of these ions on different electrodes reveals two distinct peak voltages. For the bare GCE, two relatively weak peaks corresponding to the oxidation of each metal are observed at potentials of −0.92 V and −0.62 V, respectively. In contrast, the ZIF-7/GCE electrode shows a slight decrease in the response currents, and the oxidation potentials shift to −0.86 V and −0.61 V, respectively. When the electrode surface is modified with PANI, the current values increase to 10.27 μA for Cd^2+^ oxidation and 2.06 μA for Pb^2+^ oxidation. Remarkably, the oxidation responses of Cd^2+^ and Pb^2+^ ions increase by 2.5 times on the ZIF-7@PANI/GCE compared to the bare GCE. These results strongly suggest that the combination of PANI and ZIF-7 creates novel pathways for the oxidation of these ions on the electrode surface. The synergy between PANI and ZIF-7 enhances the electrocatalytic activity for Cd^2+^ and Pb^2+^, resulting in higher current responses at lower oxidation potentials for these ions.

### 3.3. Optimization of Experimental Parameters

Electrochemical sensors for Pb^2+^ and Cd^2+^ ions are highly sensitive to operating conditions such as pH, deposited drop volume, incubation time, and drying time. Therefore, optimizing these detection parameters is crucial to achieve a highly efficient and reliable electrochemical sensor. Response Surface Methodology (RSM) is a statistical analysis technique that utilizes experimental data to optimize processes and improve the performance of detection systems [43]. RSM is based on a fractional factorial design, meaning that only certain possible combinations of factor levels are tested, significantly reducing the number of required experiments. The Box–Behnken design is a specific type of experimental design used in RSM [44]. In this design (BBD), each factor is generally studied at three different levels (low, centered, and high) along with three central test points to estimate quadratic effects in the model. In this study, the optimization of four control factors was achieved using the BBD approach to maximize the current response and examine interaction between independent variables. The selected factors included the pH of the medium (**X_1_**, 3–7), deposited drop volume (**X_2_**, 3–15 µL), incubation time (**X_3_**, 1–3 min), and drying time (**X_4_**, 1–3 h). The relationship between the response and independent variables is described by the multiple quadratic regression model (Equation (1)) [45]:

(1) $$Y=a_{0}+\overset{n}{\underset{i=1}{\sum }}a_{i}X_{i}+\overset{n}{\underset{i=1}{\sum }}a_{ii}X_{i}^{2}+\overset{n}{\underset{i=1}{\sum }}\overset{n}{\underset{j=i+1}{\sum }}a_{i}a_{j}X_{i}X_{j}+\epsilon$$

where Y is the predicted response, a_0_ is the constant, (X_i_ and X_j_) are the independent variables, a_i_ is the linear effect, a_ij_ is the interaction coefficient, a_ii_ is the quadratic coefficient, n is the number of optimized factors, and ϵ is the random error for uncertainties between predicted and determined data. The BBD design required 27 experimental runs, incorporating three central points to estimate quadratic effects. Table 1 outlines the experimental parameters along with their respective levels.

The uncoded unit regression equations for the two responses (Cd^2+^ (Equation (2)) and Pb^2+^ (Equation (3))) are given as follows:

(2) $${\hat{I}}_{Cd^{2+}}=-9.84+3.81X_{1}+0.24X_{2}+1.73X_{3}+0.56X_{4}-0.44X_{1}*X_{1}-0.017X_{2}*X_{2}\;\;\;\;\;-0.21X_{3}*X_{3}-0.15X_{4}*X_{4}+0.0018X_{1}*X_{2}+0.078X_{1}*X_{3}\;\;\;\;\;+0.122X_{1}*X_{4}-0.035X_{2}*X_{3}+0.067X_{2}*X_{4}-0.60X_{3}*X_{4}$$

(3) $${\hat{I}}_{Pb^{2+}}=-24+8.68X_{1}+0.91X_{2}+1.73X_{3}+4.31X_{4}-1.02X_{1}*X_{1}-0.021X_{2}*X_{2}\;\;\;\;\;\;\;\;\;+0.44X_{3}*X_{3}-0.54X_{4}*X_{4}-0.0076X_{1}*X_{2}+0.064X_{1}*X_{3}+0.36X_{1}\;\;*X_{4}-0.15X_{2}*X_{3}-0.086X_{2}*X_{4}-1.32X_{3}*X_{4}$$

**Figure 6:**
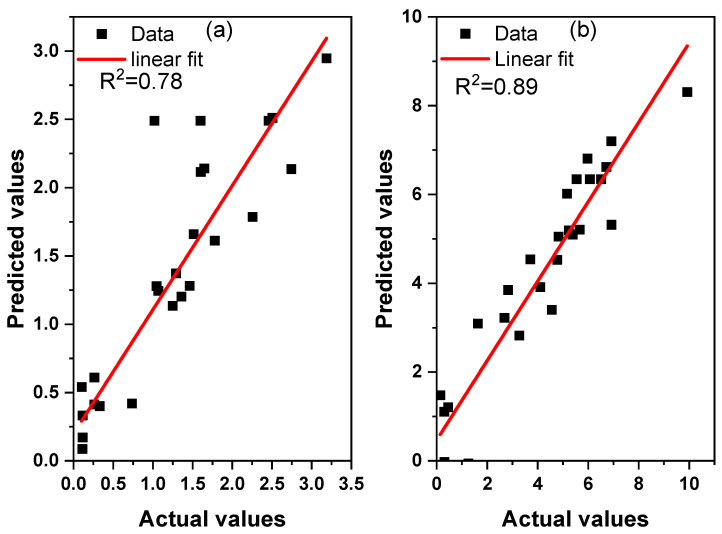
Statistical analysis fit of the RSM model (**a**) for Cd^2+^ current response and (**b**) for Pb^2+^ current response.

The statistical analysis results in Table 2 and Figure 6 demonstrate that both models $\left({\hat{I}}_{Cd^{2+}}\;\mathrm{and}\;{\hat{I}}_{Pb^{2+}}\right)$ have high R^2^ values and, thus, an excellent overall fit to the data. The values of $R_{Adj}^{2}$ suggest that both prediction models are reasonably robust. The performance of two models on new unseen data is reasonably good.

ANOVA analysis was also conducted for each model (Table 3), revealing high F-values and low *p*-values (<0.05) for both, indicating strong statistical significance. The ANOVA table summarizes linear terms, squared terms, and interactions terms. The *p*-values of the linear terms (X_1_.._4_) show that pH (X_1_) is the only significant factor. Regarding the square terms, only X_1_ ∗ X_1_ is statistically significant for both models while the square term X_2_ ∗ X_2_ is significant for the 1st model. For interaction terms, the X_3_ ∗ X_4_ (*p* < 0.05) interaction was the only statistically significant term, indicating an interaction between incubation time (X_3_) and drying time (X_4_).

**Figure 7:**
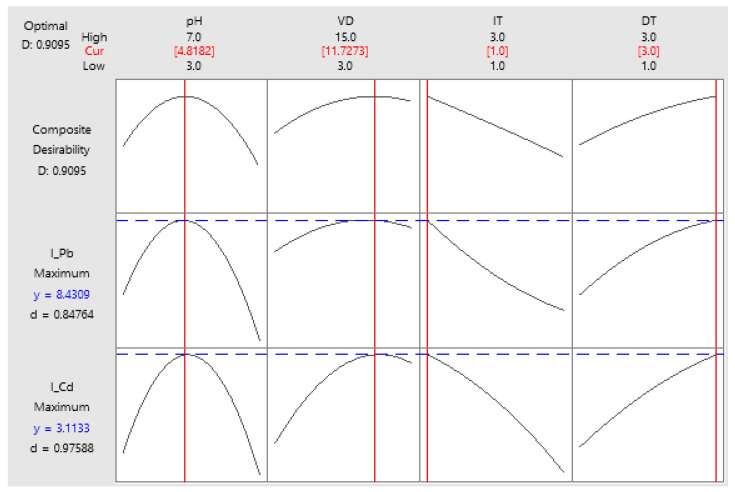
Optimization plot using desirability function.

The desirability function approach was used to optimize the heavy metal sensor by determining the ideal peak current values for Cd^2+^ and Pb^2+^ ions. The response variable (peak current for both ions) was set to “maximum” to attain the best sensor performance or desirability value, while the desired operational parameters (pH, deposited drop volume, incubation time, and drying time) were chosen within the investigated experimental range. Based on the response objective, this methodology was applied to maximize the overall desirability function. Regression analysis identified the optimal conditions as pH = 4.8, DV drop volume = 11.73 µL, incubation time IT = 1 min, and drying time DT = 3 h (Figure 7). The optimal pH of 4.8 for the simultaneous electrochemical detection of Pb^2+^ and Cd^2+^ is supported by electrochemical principles. At this slightly acidic pH, both metal ions remain in their electroactive forms, preventing precipitation as hydroxides, which occurs at higher pH levels [46]. Lower pH values can lead to competition from H^+^ ions, reducing detection sensitivity by interfering with the adsorption of metal ions onto the electrode surface [47]. Additionally, pH 4.8 minimizes interferences from other species in complex matrices, ensuring selective detection of Pb^2+^ and Cd^2+^ [48]. Research indicates that the optimal pH range for detecting these ions typically falls between 4.5 and 5.0, where both ions remain soluble and electroactive [49]. Furthermore, modified electrode surfaces at this pH facilitate efficient adsorption of these ions through favorable interactions with functional groups on the electrode material, enhancing the electrochemical response and improving sensitivity [50].

Under these ideal operating conditions, the RSM model estimated a response of 8.43 µA for Pb^2+^ ions and 3.11 µA for Cd^2+^ ions. According to Roslan et al. [51], a model is considered reliable when the desirability value approaches unity. The model’s acceptability and applicability were confirmed by a desirability of 0.9095.

An experiment was carried out under the optimized conditions (pH = 4.8, DV = 11.73 µL, IT = 1 min, and DT = 3 h) to verify the predicted responses for Cd^2+^ and Pb^2+^ ions. To ensure accuracy, experimental errors were minimized, and the RSM model’s predictions were validated, the experiment was repeated three times under these optimal conditions. As shown in Table 4, the response for Cd^2+^ ions is 1.9658 µA while the response for Pb^2+^ ions is 8.4699 µA under the optimized conditions.

### 3.4. Electrochemical Determination of Cd^2+^ and Pb^2+^ Ions

**Figure 8:**
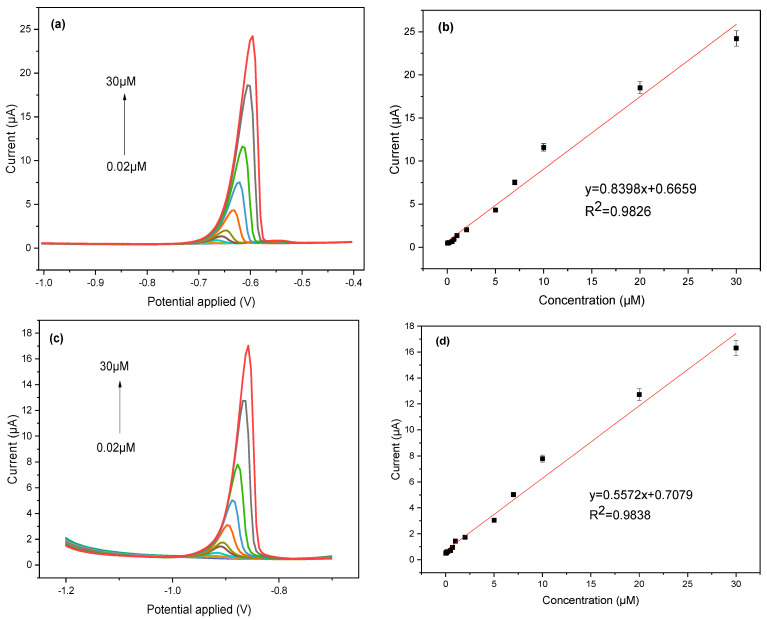
DPV curves of the ZIF-7@PANI/GCE sensor for the individual detection of (**a**) Pb^2+^ and (**c**) Cd^2+^ ions at different concentration ranges from 0.02 µM to 30 µM. Calibration curves of (**b**) Pb^2+^ and (**d**) Cd^2+^.

Using the optimal experimental parameters recommended by the model, the distinct detection capability of the ZIF-7@PANI/GCE sensor for Pb^2+^ and Cd^2+^ ions was investigated through DPV measurements. As illustrated in Figure 8, the dissolution peaks current increased linearly with the rising concentration of the target heavy metal ions. The peak current for Pb^2+^ shows a linear correlation with concentrations ranging from 0.002 µM to 30 µM, following the regression equation Ip (μA) = 0.8398 C (µM) + 0.6659 (R^2^ = 0.9826). Similarly, Figure 8b,d depicts a linear calibration curve for Cd^2+^ over the same concentration range, described by the equation Ip (μA) = 0.5572 C (µM) + 0.7079 (R^2^ = 0.9838). These results confirm the ZIF-7@PANI/GCE sensor’s high sensitivity and effective detection capabilities for Pb^2+^ and Cd^2+^ ions. This enhanced performance is attributed to the significant specific surface area and structure of the ZIF-7 matrix, which provides numerous active sites for ion interaction. Additionally, the ZIF-7@PANI composite benefits from the synergistic effects of PANI and ZIF-7, where PANI facilitates the electron transport process and the abundant -COO^−^ groups in ZIF-7 promote the pre-enrichment of Pb^2+^ and Cd^2+^ on the electrode surface.

**Figure 9:**
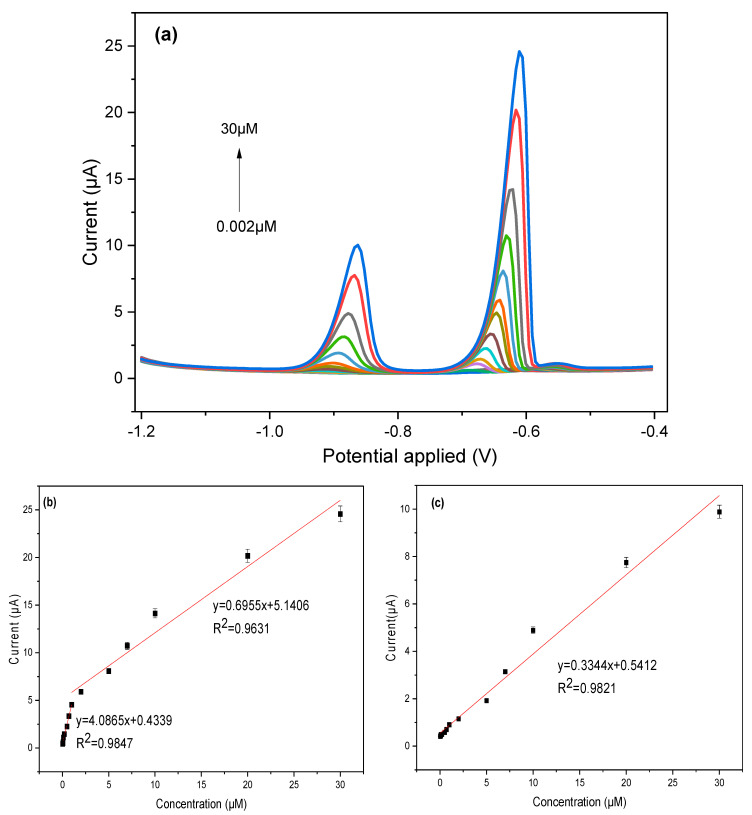
(**a**) DPV curves of ZIF-7@PANI/GCE sensor for the simultaneous analysis of Pb^2+^ and Cd^2+^ across concentrations from 0.002 µM to 30 µM. The calibration curves of (**b**) Pb^2+^ and (**c**) Cd^2+^.

A simultaneous analysis of Cd^2+^ and Pb^2+^ was conducted under the optimized conditions using the ZIF-7@PANI/GCE sensor, with a 1 min accumulation time. The tested ion concentrations ranged from 0 to 30 µM. As shown in Figure 9a, the DPV responses of Pb^2+^ and Cd^2+^ increased progressively with rising ion concentrations. Calibration curves for Pb^2+^ and Cd^2+^ were subsequently plotted, as illustrated in Figure 9b and Figure 9c, respectively. For Pb^2+^ detection, the analysis revealed two distinct sections, likely due to different adsorption behavior at low and high concentrations on the ZIF-7@PANI/GCE sensor surface. Notably, Pb^2+^ ions exhibited higher sensitivity at lower concentrations, suggesting that metal ions initially interact with the most active sites, while at higher concentrations, they progressively occupy less active sites [52]. The regression equations for Pb^2+^ were Ip (µA) = 4.0865 C (µM) + 0.4339 (R^2^ = 0.9847) within the 0.002–1 µM range and Ip (μA) = 0.6955C (µM) + 5.1406 (R^2^ = 0.9631) for 1–30µM (Figure 9b). For Cd^2+^, a single linear response was observed over the entire concentration range, following the equation Ip (µA) = 0.3344 C (µM) + 0.5412 (R ^2^ = 0.9821) from 0.02 µM to 30 µM (Figure 9c). The limits of detection (LOD) were calculated using the 3S/N method, yielding values of 2.93 nM for Pb^2+^ and 10.6 nM for Cd^2+^, confirming the sensor’s high sensitivity and suitability for trace metal ion detection.

The performance of the sensor ZIF-7@PANI/GCE was compared with other sensors for the simultaneous determination of Pb^2+^ and Cd^2+^ ions. The results, summarized in Table 5, demonstrate the good electroanalytical capabilities of the sensor developed in this study.

### 3.5. Interference Study

**Figure 10:**
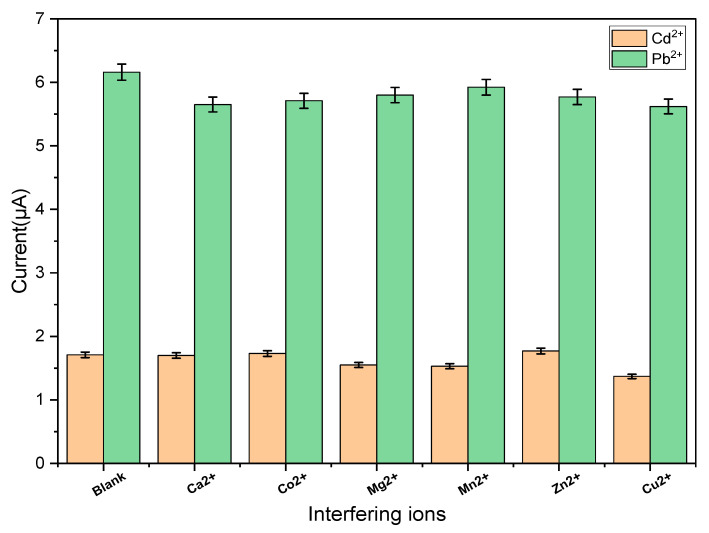
The specificity of the ZIF-7@PANI/GCE sensor toward 5 μM Cd^2+^ and Pb^2+^ against 50 µM Ca^2+^, Co^2+^, Mg^2+^, Mn^2+^, Zn^2+^, and Cu^2+^.

In real water samples, multiple ions coexist, potentially interfering with detection results. To assess the anti-interference capacity of the ZIF-7@PANI/GCE sensor, interfering ions such as Ca^2+^, Co^2+^, Mg^2+^, Mn^2+^, Zn^2+^, and Cu^2+^ were introduced at concentrations five times higher than a 5 μM Cd^2+^ and Pb^2+^ solution. As depicted in Figure 10, the current response for both lead and cadmium ions remains largely unaffected in the presence of these ions, except for Cu^2+^. This substantial interference from Cu^2+^ can be attributed to its strong competition for adsorption sites on the electrode surface, which reduces the availability of these sites for Cd^2+^ ions. Notably, the fivefold higher concentration of Cu^2+^ resulted in a significant 19% decline in the response current for Cd^2+^. In contrast, the impact of other ions was relatively minor, with reductions ranging from 0.5% to 9% for Pb^2+^ and 3% to 8% for Cd^2+^. These findings confirm that the ZIF-7@PANI/GCE sensor exhibits a good anti-interference capacity, maintaining reliable detection even in the presence of high concentrations of interfering species.

### 3.6. Real Sample

To assess the real-world applicability of the proposed sensor and evaluate its electroanalytical performance, the ZIF-7@PANI/GCE electrochemical sensor was used to quantify Pb^2+^ and Cd^2+^ in real water samples, including tap water, mineral water, and seawater from the Monastir region. The water samples were filtered to remove impurities, and initial analyses detected no signals for Pb^2+^ and Cd^2+^, indicating their absence. To validate the sensor’s performance, known concentrations of Pb^2+^ and Cd^2+^ ions were added to the samples, followed by electrochemical measurements using the ZIF-7@PANI/GCE sensor. The results, summarized in Table 6, revealed recovery rates ranging from 85% to 110.1%, confirming the sensor’s accuracy and reliability for detecting Pb^2+^ and Cd^2+^ ions in environmental water samples.

## 4. Conclusions

This study presents a high-performance modified electrode based on ZIF-7@PANI for the simultaneous detection of Cd^2+^ and Pb^2+^ ions in water samples. The ZIF-7@PANI nanocomposite was designed to leverage the excellent electrical conductivity of polyaniline (PANI) alongside the enhanced ZIF-7 nanomaterials for trace metal ion detection. The sensor’s performance was further optimized by tuning different experimental parameters, including accumulation time, deposited quantity, and drying time, using the Box–Behnken experimental design. Although pH influences sensor performance, it was controlled prior to measurements. The synergistic combination of PANI and ZIF-7 significantly improved the sensor’s sensitivity, enabling the detection of Pb^2+^ and Cd^2+^ ions at ultra-trace levels, with detection limits of 2.96 nM for Pb^2+^ and 10.6 nM for Cd^2+^. The developed sensor demonstrated outstanding selectivity, achieving strong recovery rates (85–110.1%) in real water samples. In summary, the ZIF-7@PANI/GCE sensor proves to be a promising tool for heavy metal detection, offering a reliable and efficient solution for environmental monitoring and public health protection.

## Figures and Tables

**Table 1:** Levels of the experimental parameters for the Box–Behnken design.

Parameters	Symbol	Parameter Level		
		Low (−1)	Medium (0)	High (+1)
pH	X_1_	3	5	7
Drop volume (µL)	X_2_	3	9	15
Incubation time (min)	X_3_	1	2	3
Drying time (h)	X_4_	1	2	3

**Table 2:** Statistical analysis of both model (${\hat{I}}_{Cd^{2+}}\;\mathrm{and}\;{\hat{I}}_{Pb^{2+}}$).

	$R^{2}$	$R_{Adj}^{2}$	$R_{Pred}^{2}$
${\hat{I}}_{Cd^{2+}}$	78%	52.3%	47.3%
${\hat{I}}_{Pb^{2+}}$	88.93%	76.01%	36.33%

**Table 3:** Analysis of variance of both models.

Source	DF	$\mathrm{Model}\;{\hat{I}}_{Cd^{2+}}$				$\mathrm{Model}\;{\hat{I}}_{Pb^{2+}}$			
		SS	MS	F-Value	*p*-Value	SS	MS	F-Value	*p*-Value
Model	14	22.3247	1.5946	8.73	0.000 **	155.690	11.12	6.89	0.001 **
X_1_	1	0.9391	0.9391	5.14	0.043 *	28.170	28.17	17.44	0.001 **
X_2_	1	0.0095	0.0095	0.05	0.824	0.143	0.142	0.09	0.771
X_3_	1	0.6675	0.6675	3.66	0.080	0.388	0.388	0.24	0.633
X_4_	1	0.0037	0.0037	0.02	0.889	3.900	3.899	2.41	0.146
X_1_ ∗ X_1_	1	16.0796	16.2956	89.26	0.000 **	102.381	89.29	55.29	0.000 **
X_2_ ∗ X_2_	1	1.7649	2.0175	11.05	0.006 **	3.493	3.113	1.93	0.190
X_3_ ∗ X_3_	1	0.1557	0.2368	1.30	0.277	2.272	1.015	0.63	0.443
X_4_ ∗ X_4_	1	0.1183	0.1183	0.65	0.437	1.541	1.540	0.95	0.348
X_1_ ∗ X_2_	1	0.0019	0.0019	0.01	0.921	0.034	0.033	0.02	0.888
X_1_ ∗ X_3_	1	0.0969	0.0969	0.53	0.480	0.066	0.066	0.04	0.843
X_1_ ∗ X_4_	1	0.2396	0.2396	1.31	0.274	2.107	2.106	1.30	0.276
X_2_ ∗ X_3_	1	0.1793	0.1793	0.98	0.341	3.204	3.203	1.98	0.184
X_2_ ∗ X_4_	1	0.6373	0.6373	3.49	0.086	1.063	1.062	0.66	0.433
X_3_ ∗ X_4_	1	1.4315	1.4315	7.84	0.016 *	6.930	6.929	4.29	0.061
Lack-of-Fit	10	2.1053	0.2105	4.93	0.180	19.336	1.933	84.85	0.012 *
Pure Error	2	0.0854	0.0427			0.046	0.022		
Total	26	24.5154				175.071			

DF: degree of freedom, SS: sum of squares, MS: mean of squares, * significant at 5%, ** significant at 1%.

**Table 4:** Results of experimental check of the predicted response for both ions at optimal conditions.

	pH	VD (µL)	IT (min)	DT (h)	$I_{Cd^{2+}}\left(µA\right)$	$I_{Pb^{2+}}\left(µA\right)$
Predicted	4.8	11.73	1	3	3.11 ± 0.78	8.43 ± 2.32
Experimental	4.8	11.73	1	3	1.9658	8.4699

**Table 5:** Comparison of the electroanalytical performance of different sensors used for the simultaneous detection of Cd^2+^ and Pb^2+^ ions.

Materials	Method	LOD (nM)		Linear Range (µM)		References
		Pb^2+^	Cd^2+^	Pb^2+^	Cd^2+^	
CUiO-66/Bi/GCE	SWASV	5.98	41.57	0.178–1.067	0.178–1.067	[53]
Bi/GDY/GCE	DPASV	0.146	0.171	0.010–100.0	0.010–100.0	[54]
UiO-66-NH_2_/rGO	DPASV	16.39	-	0.2–3.2	-	[55]
UiO-66-NH_2_/GaOOH	DPASV	16	28	0.55–2.50	0.35–1.60	[56]
Pd NPs-porous carbon/GCE	DPV	50	41	0.5–8.9	0.5–5.5	[57]
ZIF-7@PANI/GCE	DPV	2.96	10.6	0.002–11–30	0.02–30	This work

SWASV: square wave stripping anodic voltammetry, GCE: glassy carbon electrode, DPASV: differential pulse anodic stripping voltammetry, CUiO-66: carbonized Zr-MOF, Bi: bismuth, GDY: graphdiyne, rGO: reduced graphene oxide, DPV: differential pulse voltammetry, Pd NPs: palladium nanoparticles.

**Table 6:** Determination of the content of Cd^2+^ and Pb^2+^ ions in tap water, mineral water, and seawater from the Monastir region using the ZIF-7@PANI/GCE sensor.

Samples	Metal Ions	Added (µM)	Found (µM)	Recovery (%)
Mineral water	Pb^2+^	1	0.91	91
		10	9.2	92
	Cd^2+^	1	1.01	101
		10	10.91	109.1
Tap water	Pb^2+^	1	0.85	85
		10	9.3	93
	Cd^2+^	1	0.89	89
		10	10.57	105.7
Sea water	Pb^2+^	1	0.94	94
		10	10.09	100.9
	Cd^2+^	1	1.088	108.8
		10	11.01	110.1

## Data Availability

Data are contained within the article.
